# Up-regulation of intra-tumour LDLR gene expression is associated with statin treatment and better prostate cancer prognosis

**DOI:** 10.2340/1651-226X.2025.43788

**Published:** 2025-10-07

**Authors:** Kia Eistrup Fonfara, Jacob Fredsøe, Benedicte Parm Ulhøi, Signe Borgquist, Michael Borre, Karina Dalsgaard Sørensen

**Affiliations:** a Department of Molecular Medicine, Aarhus University Hospital & Department of Clinical Medicine, Aarhus University, Aarhus, Denmark; b Department of Pathology, Aarhus University Hospital, Aarhus, Denmark; c Department of Oncology, Aarhus University Hospital, Aarhus, Denmark; d Department of Urology, Aarhus University Hospital, Aarhus, Denmark

**Keywords:** atorvastatin, simvastatin, cholesterol, differential expression analysis, low-density lipoprotein receptor

## Abstract

**Background:**

Several studies have reported associations between statin treatment and a more favourable prognosis in prostate cancer (PC) patients. The underlying biology, however, has not been fully investigated. Objective: To perform whole-transcriptome profiling of prostate tumour samples from PC patients to identify gene expression patterns and molecular pathways that may be associated with statin treatment. Furthermore, to investigate correlations between statin-associated gene expression changes and clinical outcomes.

**Material and methods:**

We performed messenger Ribonucleic Acid (mRNA) sequencing on radical prostatectomy specimens from 186 patients with clinically-localised PC. The final dataset included 93 statin-users (93 PC and 43 adjacent normal [AN] samples) and 93 non-users (93 PC and 43 AN samples). We performed Differential Expression Analysis and Gene Set Enrichment Analysis (GSEA) between statin-users and non-users. Genes of interest were included in uni- and multivariate analyses exploring time to Biochemical Recurrence (BCR).

**Results:**

Comparing statin-users and non-users, there were zero significantly differentially expressed genes (DEGs) in AN samples and 163 DEGs in PC samples. In statin-users, GSEA revealed downregulation of pathways known to drive PC aggressiveness, most significantly epithelial-mesenchymal transition. Low-density Lipoprotein Receptor (LDLR) was among the top-upregulated genes and expressed higher in atorvastatin than in simvastatin users. The LDLR upregulation was associated with prolonged BCR-free survival.

**Interpretation:**

We identified several genes and pathways in PC tissue potentially associated with the reported beneficial effects of statin treatment in PC. Specifically, we identified an association between statin treatment and intra-tumour LDLR upregulation. This study contributes to the understanding of statin-mediated effects on PC.

## Graphical abstract

**Figure:**
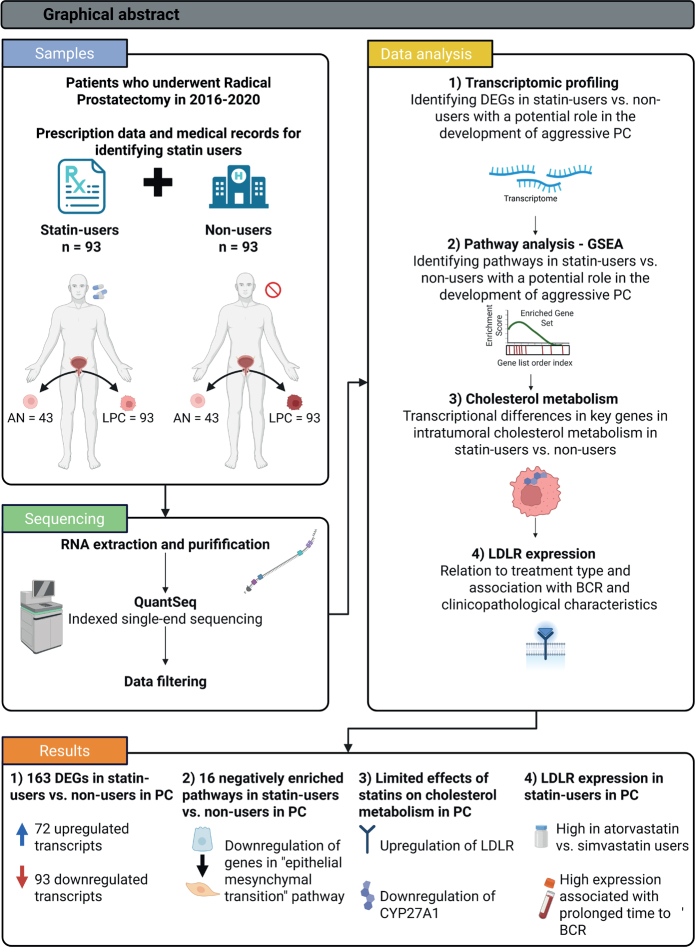

## Introduction

### Prostate cancer

Prostate cancer (PC) is the most common cancer in men in the Nordic countries [1]. The choice of treatment depends on the clinicopathological characteristics (Tumor-Node-Metastasis (TNM) stage, Gleason score, serum prostate-specific antigen [PSA] levels), and individual factors such as age and comorbidity [2]. Early-stage (localised) PC may be cured by radical prostatectomy (RP) or by radiation therapy. Alternatively, low-risk early-stage PC may be managed safely by active surveillance. Nevertheless, some patients progress to incurable advanced/metastatic PC. At this point, tumour growth may be suppressed by androgen deprivation therapy (ADT) as PC cells usually require androgen stimulation to grow [2]. In line with this, the role of cholesterol (precursor molecule to androgen) in PC disease biology has attracted growing interest. In large epidemiological studies, the use of statins (cholesterol-lowering drugs) has been associated with lower PC risk and lower mortality in PC patients [3].

### Statin treatment and cancer

Statins are prescribed to patients with high cholesterol to reduce the risk of cardiovascular disease by lowering plasma cholesterol levels. More specifically, statins block the 3-hydroxy-3-methyl-glutaryl-CoA (HMG-CoA) reductase and thereby *de novo* intracellular synthesis of cholesterol. This leads to an upregulation of low-density lipoprotein (LDL) receptors, predominantly in liver cells, mediating an increased uptake of cholesterol from plasma. The biological effect of statin drugs on PC remains elusive. Conceivably, such effects could be indirectly caused by the systemic lowering of blood cholesterol levels or directly by blocking the HMG-CoA reductase in prostate (cancer) cells.

Several prior studies have reported that high-fat diets and high blood cholesterol levels are associated with an increased risk of PC and disease progression [4]. Furthermore, it has been reported that statins may accumulate in the prostate gland and that direct inhibition of the HMG-CoA reductase in prostate cells may lower the levels of intracellular cholesterol and several intermediate compounds, in turn inhibiting PC cell proliferation and survival [5]. Hence, the potential effects of statin on PC may be direct, indirect, or a combination of both.

To further elucidate the possible molecular links between statin treatment and PC pathobiology, we firstly performed genome-wide expression analyses of adjacent normal (AN) and PC tissue samples from patients who were statin and non-statin users. Secondly, we analysed intra-tumour expression patterns of key genes in the cholesterol metabolism as well as possible associations with serum cholesterol levels, to further explore local and systemic regulatory factors. Lastly, we analysed the correlation with clinicopathological characteristics known to be linked with more aggressive PC and performed biochemical recurrence (BCR)-free survival analyses.

## Patients/material and methods

### Patient population and tumour RNA sequencing (QuantSeq)

The cohort comprised 190 patients who had undergone curatively-intended RP for clinically-localised PC at Aarhus University Hospital between 2016 and 2020, and for whom archived tumour tissue samples and statin-use data were available. Statin-users and non-users were identified using the Danish National Prescription Registry [6]. Statin-use was validated in pre-surgery medical records, to confirm that the prescribed drug was used at the time of surgery. A total of 13 patients (6%) were not selected for sequencing due to discrepancy between prescription records and medical records. Statin-users were defined as having redeemed at least two prescriptions of either simvastatin or atorvastatin (the most commonly used statins in Denmark during the study period) at any time before RP and being registered as a current statin-user in the hospital medical records at the time of the operation. Non-users were defined as having redeemed no prescriptions of statins of any kind and having no record of statin use in the hospital records. In total, we identified 97 statin-users and 93 non-users and retrieved formalin-fixed, paraffin-embedded (FFPE) RP specimens from the Department of Pathology, Aarhus University Hospital, Denmark, for all 190 patients.

Prior to RNA extraction from RP specimens, an expert pathologist marked a representative PC area (highest Gleason score). For a random subset of patients (*n* = 94, including 49 users and 45 non-users), a representative AN area was also marked. Two 1.5 mm. tissue cylinders were punched out from each marked area, and RNA extraction was performed using the RNeasy FFPE Kit (Qiagen) as recommended by the manufacturer. In total, RNA was extracted from 97 PC and 49 AN samples from 97 statin-users, and 93 PC and 45 AN samples from 93 non-users.

Libraries for Next-Generation Sequencing (NGS) were constructed using the QuantSeq 3’ mRNA-Seq Library Prep Kit for Illumina (Lexogen), which uses oligo-dT priming and can provide robust expression profiles for highly fragmented RNA extracted from FFPE tissue stored for up to 10 years [7]. The FFPE blocks used here had been stored for ≤5 years before RNA extraction. Libraries were single-end sequenced on three SP flow cells (v1.5) run on the Novaseq 6,000 sequencing system (Illumina). After filtering and technical quality control (Figure S1), the final RNAseq dataset included 93 PC and 43 AN tissue samples from 93 statin-users as well as 93 PC and 43 AN samples from 93 non-users. Technical quality control criteria that led to the exclusion of samples from further analyses were: (1) too low RNA concentration (< 10 ng/µL on DropSense96), (2) too low DNA concentration after library preparation (< 0.3 nM on TapeStation), and/or (3) less than 1 million mapped reads.

Clinicopathological characteristics for the final cohort are described in Table 1. Furthermore, details on statin use history and blood cholesterol levels prior to surgery are presented in Table S1. Information on patient cholesterol levels was extracted from the regional hospital laboratory system (LABKA II). For each patient, total plasma cholesterol (P-Cholesterol) and plasma low-density lipoprotein (P-LDL) levels were calculated based on the mean values from all blood samples drawn 5 years before RP. High P-cholesterol was defined as ≥ 5 mmol/L and high P-LDL as ≥ 3 mmol/L, according to clinical guidelines [8].

**Table 1:** Patient clinical characteristics.

Statin treatment	Non-user	Statin-user (Total)	Statin-user (Atorvastatin)	Statin-user (Simvastatin)
**Patients**				
*n*	93	93	45	48
**Age, years**				
Median	68.1	69.7	69.7	69.6
(IQR)	(61.2–71.4)	(66.2–71.9)	(66.6–71.9)	(65.0–71.8)
Unknown	0 (0%)	0 (0%)	0 (0%)	0 (0%)
**PSA**				
Median ng/mL	8.7	9.1	7.9	10.2
(IQR)	(6.0–13.2)	(5.7–14)	(5.9–12.6)	(5.7–14.5)
Unknown	0 (0%)	0 (0%)	0 (0%)	0 (0%)
**Pathological ISUP Grade Group**				
1	13 (13.9%)	4 (4.3%)	2 (4.4%)	2 (4.1%)
2	56 (60.2%)	63 (67.7%)	30 (66.7%)	33 (68.8%)
3	11 (11.8%)	12 (12.9%)	6 (13.3%)	6 (12.5%)
4	1 (1.1%)	8 (8.6%)	4 (8.9%)	4 (8.3%)
5	4 (4.3%)	6 (6.4%)	3 (6.7%)	3 (6.2%)
Unknown	8 (8.6%)	0 (0%)	0 (0%)	0 (0%)
**Pathological T stage**				
T2	48 (51.6%)	50 (53.7%)	26 (57.8%)	24 (50%)
T3	33 (40.2%)	42 (45.2%)	19 (42.2%)	23 (47.9%)
T4	1 (1.1%)	0 (0%)	0 (0%)	0 (0%)
Unknown, *n*	11 (11.8%)	1 (1.1%)	0 (0%)	1 (2.1%)
**Surgical margin status**				
Positive	28 (55.9%)	29 (66.7%)	14 (31.1%)	15 (31.3%)
Negative	52 (30.1)	62 (31.2%)	31 (68.8%)	31 (64.5%)
Unknown	13 (14.0%)	2 (2.2%)	0 (0%)	2 (4.2%)
**CAPRA-S risk Nomogram**				
Low (0–2)	28 (30.1%)	25 (26.9%)	13 (28.9%)	12 (25%)
Intermediate (3–5)	37 (39.8%)	42 (45.2%)	22 (48.9%)	20 (41.6%)
High (≥ 6)	15 (16.1%)	25 (26.8%)	10 (22.2%)	15 (31.3%)
Unknown	13 (14%)	1 (1.1%)	0 (0%)	1 (2.8%)
**Biochemical recurrence status**				
Recurrence	10 (10.8%)	30 (32.2%)	14 (31.1%)	16 (33.3%)
No recurrence	80 (86%)	62 (66.7%)	30 (66.6%)	32 (66.7%)
Unknown	3 (3.2%)	1 (1.1%)	1 (2.2%)	0 (0%)
**Follow-up time**				
Months	23.4	35.9	29.8	43.4
(IQR)	(17.5–25.4)	(24.3–48.3)	(20.9–39.7)	(30.5–50)
Unknown	3 (3.2%)	1 (1.1%)	1 (2.2%)	0 (0%)

IQR: interquartile range; NA: not applicable; PSA: prostate-specific antigen.

Data is *n* (%) or median (IQR).

### Data management and statistical analysis

All statistical analyses were performed using R version 4.3.2 [9] and Rstudio version 2023.06.1 [10]. Salmon version 1.4.0 was used to map reads to the Genome Reference Consortium Human Build 38 (hg38) and to quantify indexed reads. Transcripts with < 10 counts per million reads (CPM) in <20% of all samples were discarded. Normalisation, correction for batch effect, and differential expression analysis were performed using the R packages, edgeR version 4.0.3 and limma version 3.58.1. *P* values < 0.05 were considered statistically significant. The Benjamini-Hochberg (BH) procedure was used to correct for multiple testing.

Gene Set Enrichment Analysis (GSEA) was used for exploring molecular pathways enriched in statin-users, and performed using the R package fgsea and the Hallmark gene sets (S) from MSigDB [11, 12]. The pre-ranked list of genes used as input for analysis was based on all differentially expressed genes (DEGs) between statin-users and non-users, ranked according to log2 fold change (logFC).

We used the Wilcoxon rank-sum test to assess associations between statin use and expression of preselected key cholesterol and androgen receptor (AR) signalling genes based on existing literature [13]. Spearman’s correlation was used to assess links between low-density lipoprotein receptor (LDLR) and AR-related genes.

Associations between LDLR expression, statin type, dosage, treatment length, P-LDL levels, and clinicopathological characteristics were tested using Wilcoxon rank-sum, Kruskal-Wallis, and Spearman’s correlation tests using the stats (v4.3.2) package.

Cox regression analyses were performed for statin-user status, P-LDL level, and LDLR expression level using the R packages ‘survival’ and ‘survminer’ with BCR as endpoint. Kaplan-Meier analysis assessed BCR-free survival by LDLR expression in statin users and non-users. Patients without BCR events were censored at their last follow-up. The common closing date for BCR events was 01 March 2022. Patients were stratified into low and high LDLR expression using a cut-off value obtained by Youden’s J statistic with the pROC package. In multivariate Cox regression analyses, we adjusted for CAPRA-S risk score – defined as low-risk (0–2), intermediate-risk (3–5), and high-risk (≥ 6). Patients were excluded from multivariate analysis if the CAPRA-S risk group could not be calculated due to missing clinical information.

Multidimensional scaling and GSEA were plotted using limma version 3.58.1 and fgsea, respectively. Other data were visualised with R package ggplot2 version 3.4.4 and ggpubr 0.6.0.

## Results

### Transcriptome profiling of prostate tumour samples identified dysregulated genes associated with pre-surgery statin use

To investigate if statin treatment is associated with gene expression patterns in primary tumour tissue samples from PC patients, we performed mRNA sequencing (QuantSeq) on RP tissue samples from 93 statin-users and 93 non-users. Statin-users had a median treatment time of 8.7 years (IQR [interquartile range] = 4.5–11.9 years), with a median treatment dose of 30.2 mg/day (IQR = 20.4–38.0 mg/day) (Table S1). Non-users had no history of statin use in hospital medical journals and had not retrieved a prescription of statin at least 12 years prior to RP. In total, 75.23% (70/93) of statin-users had clinically defined low total P-Cholesterol (<5 mmol/L) compared to 35.5% (33/93) of non-users (Table S1).

The final RNAseq dataset that passed quality control included 90 PC and 43 AN samples from 93 statin-users, and 90 PC and 43 AN tissue samples from 93 non-users (Figure S1, see Materials and methods for details). Principal component analysis (PCA) showed an overall separation between AN and PC samples across the full cohort (Figure S2), supporting the validity of our results. There was no distinct clustering according to statin user status for PC nor AN tissue samples (Figure S2), consistent with the hypothesis that gene expression differences associated with statin-use are likely more subtle than expression differences associated with malignant transformation.

In line with this, edgeR analysis identified 8,459 significantly (*p* < 0.05, (BH)-adjusted) DEGs, when comparing AN and PC samples across the full cohort (52% of the 16,190 genes analysed). In contrast, when comparing AN prostate tissue samples from statin-users and non-users, we found zero (0%) significantly DEGs (*p* < 0.05, BH- adjusted; data not shown), indicating no or negligible effects of statin use on gene expression patterns in AN prostate tissue samples in this cohort. However, a total of 163 genes (1.0%) were significantly differentially expressed in PC tissue samples from statin-users compared to non-users, including 72 upregulated and 93 downregulated genes associated with systemic statin use (*p* < 0.05, BH-adjusted). The top 30 most significantly DEGs are listed in Table 2, and include several genes previously reported as dysregulated in PC and/or other cancer types as well as several genes (*COA7*, *MIEF2*, *LDLR*, and *GRHPR*) with known roles in cellular metabolism (Table S2). While some of the observed differences in intra-tumour gene expression levels may reflect PC molecular heterogeneity, it is also feasible that the observed changes reflect the direct/indirect effects of statin treatment on specific genes. Indeed, one of the most significantly upregulated genes in PC tissue samples in statin-users compared to non-users was *LDLR* (LogFC = 0.416, *p* = 5.37E-05, Table 2).

**Table 2:** Top 30 differentially expressed genes.

Gene	*P*. val	*P*. adj	LogFC	Nomenclature
**Downregulated in statin-users vs. non-users**				
*ZNF853*	5.93E-08	0.001	–0.870	Zinc Finger Protein 853
*DCAF12L2*	1.43E-06	0.006	–1.123	DDB1 And CUL4 Associated Factor 12 Like 2
*ZNF800*	1.48E-06	0.006	–0.291	Zinc Finger Protein 800
*NEURL1B*	2.72E-06	0.009	–0.545	Neuralized E3 Ubiquitin Protein Ligase 1B
*RBPJ*	3.33E-06	0.009	–0.191	Recombination Signal Binding Protein For IG Kappa J Region
*FOXO1*	4.72E-06	0.011	–0.451	Forkhead Box O1
*TSC22D3*	1.42E-05	0.023	–0.493	TSC22 Domain Family Member 3
*SNHG6*	1.54E-05	0.023	–0.336	Small Nucleolar RNA Host Gene 6
*DOCK11*	1.59E-05	0.023	–0.740	Dedicator Of Cytokinesis 11
*PPP4R2*	2.86E-05	0.027	–0.223	Protein Phosphatase 4 Regulatory Subunit 2
*CTDSPL*	3.16E-05	0.027	–0.251	CTD Small Phosphatase Like
*AL445426.1*	4.18E-05	0.029	–0.962	–
*DEK*	5.21E-05	0.030	–0.199	DEK Proto-Oncogene
*COL17A1*	5.98E-05	0.030	–1.282	Collagen Type XVII Alpha 1 Chain
**Upregulated in statin-users vs. non-users**				
*AC073225.1*	1.07E-06	0.006	0.787	–
*CCDC124*	6.56E-06	0.013	0.401	Coiled-Coil Domain Containing 124
*PGBD4*	1.9E-05	0.026	0.510	PiggyBac Transposable Element Derived 4
*GOLGA6L2*	2.29E-05	0.027	1.343	Golgin A6 Family Like 2
*COA7*	2.75E-05	0.027	0.467	Cytochrome C Oxidase Assembly Factor 7
*RNF223*	2.85E-05	0.027	0.965	Ring Finger Protein 223
*AL354919.1*	2.9E-05	0.027	0.590	–
*AP002748.6*	3.03E-05	0.027	0.973	–
*MIR3936HG*	3.34E-05	0.027	0.594	MIR3936 Host Gene
*BCL2L14*	3.69E-05	0.027	0.778	BCL2 Like 14
*MIEF2*	3.7E-05	0.027	0.412	Mitochondrial Elongation Factor 2
*OR7E122P*	4.47E-05	0.030	1.083	Olfactory Receptor Family 7 Subfamily E Member 122
*OTUB1*	5.09E-05	0.030	0.242	OTU Deubiquitinase, Ubiquitin Aldehyde Binding 1
*RN7SL440P*	5.21E-05	0.030	0.886	–
*LDLR*	5.37E-05	0.030	0.416	Low Density Lipoprotein Receptor
*GRHPR*	6.02E-05	0.030	0.239	Glyoxylate And Hydroxypyruvate Reductase

*P*. val: *p*-value; *P*. adj: BH adjusted *p*-value; LogFC: log2 fold change; DEGs: differentially expressed genes.

Differential expression analysis with top DEGs between statin-users and non-users in PC samples based on p-value. For additional information, see Table S2.

### Dysregulated molecular pathways in prostate tumour tissue associated with statin treatment

**Figure 1:**
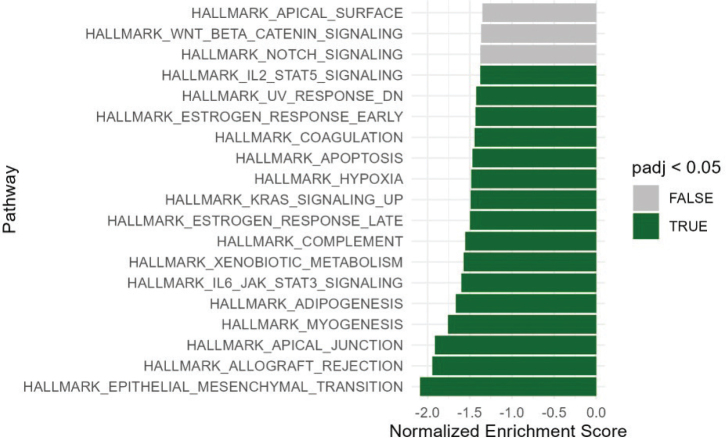
Gene set enrichment analysis. Top 20 enriched pathways based on p-value are presented. Hallmark gene sets enrichment analysis based on all 16,190 genes in statin-users versus non-users in PC samples. Green bars are significantly downregulated pathways in statin-users versus non-users. No pathways were significantly upregulated. Significance threshold = p-value < 0.05, adjusted by the Benjamin-Hochberg Procedure. P-adj: adjusted p-value; PC: prostate cancer.

To explore the biological pathways in PC tissue that may be associated with statin use, we performed GSEA based on logFCs between statin-users and non-users for all 16,190 genes analysed. Using the Hallmark gene set, GSEA identified 16 downregulated pathways (*p* < 0.05, BH-adjusted) and no significantly upregulated pathways in statin-users. Top 20 pathways based on *p*-value are presented in Figure 1. Of note, several oncogenic pathways (‘Epithelial-mesenchymal transition (EMT)’, ‘IL6-JAK-STAT3 signalling’ and ‘KRAS signalling up’) as well as inflammation-associated pathways (‘Allograft rejection’, ‘IL-STAT5-signalling’, ‘Complement’ and ‘Interferon-gamma response’) were significantly downregulated in statin-users. Moreover, while pathways related to female steroid hormone signalling (‘Estrogen response late’ and ‘Estrogen response early’) were significantly downregulated, we did not observe any significant differences in regards to male steroid hormone signalling (pathway ‘Androgen Response’; *p* > 0.05, BH-adjusted, Figure S4).

The top genes driving EMT downregulation were *GAS1, ACTA1, FLNA*, and *MYLK* (see Table S4), and *ACTA1, FLNA,* and *MYLK* are all involved in cytoskeletal changes during EMT.

### Intra-tumoral expression levels of most key cholesterol metabolism genes were similar in statin users and non-users

**Figure 2:**
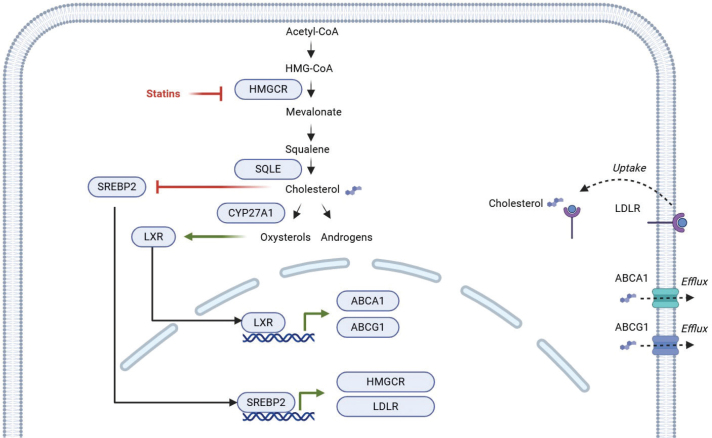
Intra-tumoral cholesterol metabolism [14, 15]. Created in BioRender. Fonfara, K. (2025) https://BioRender.com/x97t880. Cholesterol may be produced intracellularly by using the mevalonate pathway: acetyl-CoA is converted to squalene in a process controlled by the rate-limiting enzyme HMGCR, the target of statin drugs. Squalene is converted into cholesterol in a process controlled by SQLE, the second rate-limiting enzyme in cholesterol biosynthesis. Cholesterol may be used to produce e.g. androgens or converted into oxysterol by enzymes such as CYP27A1. SREBP2 is a transcription factor that activates cholesterol biosynthesis and increases uptake by upregulating HMGCR and LDLR. SREPB2 is regulated through a negative feedback mechanism by cholesterol availability. LXR is a transcription factor that inhibits LDL-mediated cholesterol uptake, suppresses cholesterol biosynthesis, and promotes cholesterol efflux mediated by ABCA1 and ABCG1. Oxysterols function as ligands for LXRs, thereby maintaining cholesterol homeostasis by reducing the amount of intracellular cholesterol. The regulation of cholesterol pathways may be dysregulated in PC. LDLR: Low-density Lipoprotein Receptor; LDL: low-density lipoprotein; PC: prostate cancer.

Our observation that LDLR was significantly upregulated in PC tissue samples from statin-users compared with non-users (Table 2) could indicate a potential statin-mediated effect on cholesterol metabolism within the tumour. Hence, we investigated expression patterns for other key genes known to be involved in cholesterol metabolism (see Figure 2, schematic model of putative cholesterol pathway, generated based on the existing literature). We observed no significant expression differences in statin-users vs. non-users (Table 3) for the rate-limiting genes in intracellular cholesterol biosynthesis (*HMGCR* and *SQLE*), nor for genes encoding transcription factors (*SREPB2* and *LXR*) or cholesterol efflux transporters (*ABCA1* and *ABCG1*). We did, however, observe significant downregulation of *CYP27A1* in statin-users (Statin-users = 3.51, non-users = 4.19, *p* = 0.024, Wilcoxon, Table 3).

**Table 3:** Intra-tumoral cholesterol metabolism.

Gene	Median expression level (logCPM)			Nomenclature
	Statin-users	Non-users	*P*. val.	
**HMGCR**	4.26	4.21	0.84	3-Hydroxy-3-Methylglutaryl-CoA Reductase
**SQLE**	4.58	4.50	0.33	Squalene Epoxidase
**LXR**	3.58	3.49	0.60	Liver X receptor
**SREBF2**	4.78	4.89	0.57	Sterol Regulatory Element Binding Factor 2
**CYP27A1**	3.51	4.19	0.024	Cytochrome P450 Family 27 Subfamily A Member 1
**ABCG1**	3.94	3.92	0.98	ATP-Binding Cassette Sub-Family G Member 1
**ABCA1**	4.24	4.29	0.79	ATP-Binding Cassette Sub-Family A Member 1
**(LDLR)**	6.99	6.58	< 0.001	Low Low-density lipoprotein Receptor

*P*. val: *p* value; logCPM: Log Counts Per Million.

Expression level of genes covering different areas of intra-tumoral cholesterol metabolism in statin-users vs. non-users compared using Wilcoxon signed-rank test.

### Intra-tumour LDLR upregulation in statin users associated with treatment type and was unrelated to plasma LDL levels

**Figure 3:**
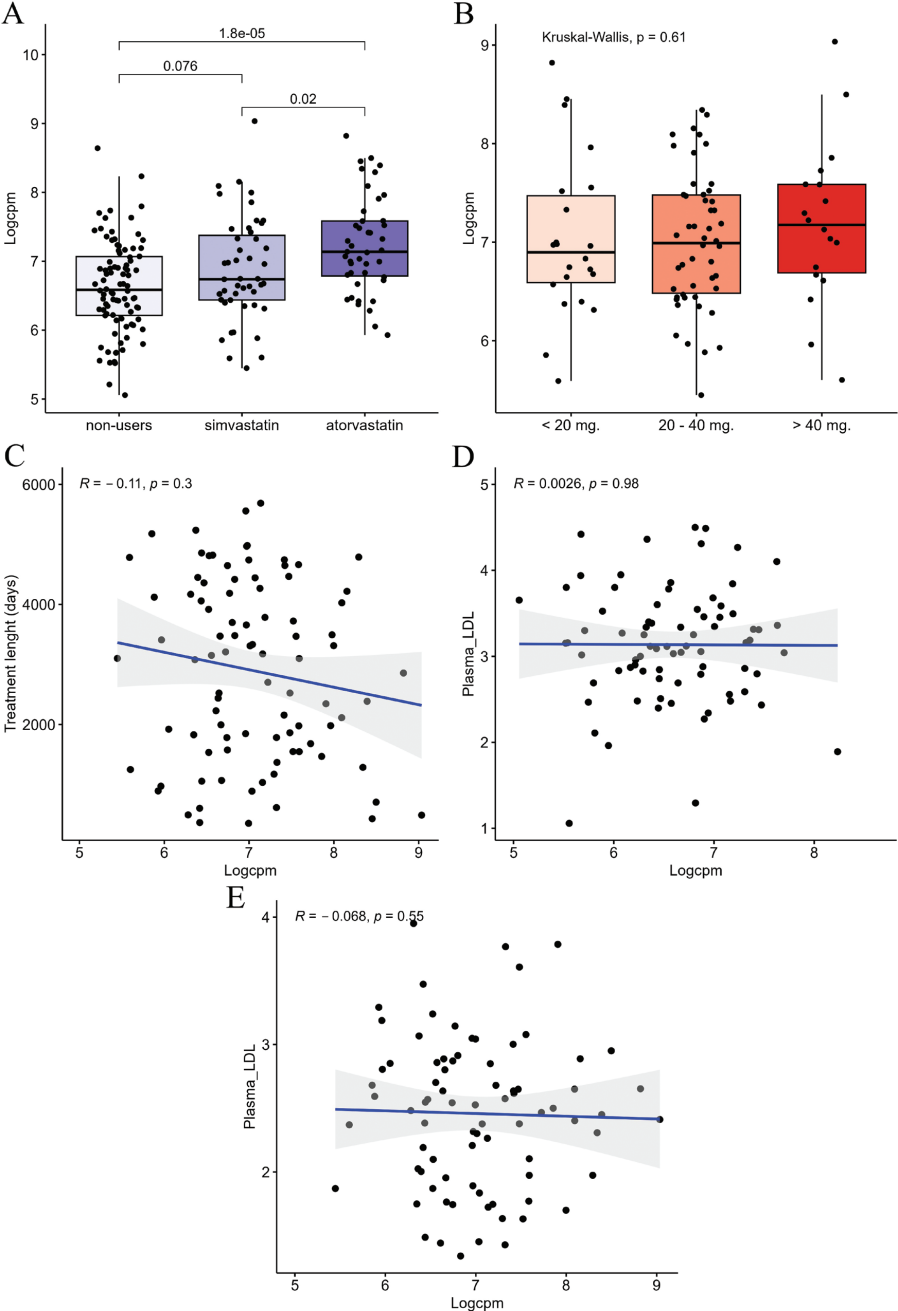
LDLR expression in PC. (A–E) LDLR expression in PC (n = 180). (A) LDLR expression in different types of statins compared using the Wilcoxon signed-rank test. P-value shown above brackets. (B) LDLR expression in patients treated with low, intermediate and high doses of statin, investigated using Kruskal-Wallis test. (C) LDLR expression correlated to treatment length using Spearman correlation. (D + E) LDLR expression correlated to plasma LDL concentration estimating the Spearman correlation coefficient in Non-users (N = 78, figure D) and Statin-users (N = 80, figure E). Patients with missing data on plasma LDL concentration before surgery were excluded from this analysis. Logcpm: Normalized log Counts per Million; R: Spearman correlation coefficient; p: p-value PlasmaLDL; Plasma LDL concentration – mmol/L. LDLR: Low-density Lipoprotein Receptor; LDL: low-density lipoprotein; PC: prostate cancer.

Next, we explored associations between intra-tumour LDLR expression, treatment type, dose, and length. We compared LDLR expression between simvastatin users (*n* = 47) and atorvastatin users (*n* = 43). We observed significant upregulation of LDLR in atorvastatin-users compared with simvastatin-users (*p* = 0.02, Wilcoxon, Figure 3A). Simvastatin treatment was only borderline associated with upregulation of LDLR compared to non-users (*p* = 0.07, Wilcoxon, Figure 3A), while atorvastatin treatment was significantly associated with LDLR upregulation (*p* = 0.000018, Wilcoxon, Figure 3A). Next, we divided statin-users into subgroups based on treatment dose (low = 0–20 mg/day, intermediate = 20–40 mg/day, and high = 40–80 mg/day). We observed no significant correlations between dose and LDLR expression, nor between treatment length and LDLR expression (*p* > 0.05, Figure 3B–C).

Prior studies have proposed that a reduction of plasma LDL in patients receiving statin treatment may have an indirect effect on intra-tumoral cholesterol metabolism [16]. In our study, we observed no significant correlation between intra-tumour LDLR expression and plasma LDL concentration in either statin-users or non-users (*p* > 0.05, Spearman’s ρ, Figure 3D–E). Our results are consistent with a possible direct effect of statin treatment on LDLR expression in prostate tumour tissue. Further mechanistic studies are warranted but beyond the scope of the current work.

## LDLR expression correlated with several key genes in AR signalling

Androgen Receptor signalling is a key target in PC and regulates cholesterol metabolism. Hence, we investigated associations between statin use, LDLR expression, and AR-related genes (*AR*, *KLK3*, coactivators *NCOA1-3*, and corepressors *NCOR1-2*). *NCOR2* was downregulated in statin users vs. non-users (*p* = 0.026, Table S3). Low-density Lipoprotein Receptor expression was borderline significantly negatively correlated with AR expression in both non-users and statin-users (*R* = –0.2 and –0.19, *p* = 0.06 and 0.07, respectively, Table S3). The AR corepressor genes (*NCOR1, NCOR2*) were negatively correlated with LDLR expression in non-users (*R* = –0.16 and –0.3, *p* = 0.13 and 0.005, respectively), and even more prominently in statin-users (*R* = –0.3 and –0.4, *p* = 0.004 and < 0.0001, respectively).

### LDLR expression is associated with prolonged time to BCR after adjusting for CAPRA-S risk group

Finally, we examined the relationship between statin-user status, intra-tumour LDLR expression, and the aggressiveness and recurrence rates of PC. We observed no significant associations between intratumor LDLR expression and Gleason Grade, T-stage, surgical margin status, BCR status, or pre-operative PSA (Figure S5).

In univariate Cox regression analysis, statin users exhibited shorter BCR-free survival compared to non-users (HR = 2.1, 95% CI = 1.03–4.58; Table 4), but this correlation did not remain significant in multivariate analysis after correction for CAPRA-S risk group (HR = 1.74, 95% CI = 0.74–3.7, Table 4).

**Table 4:** Biochemical recurrence risk.

Variable	Crude analysis			Adjusted analysis		
	HR	95% CI	*P*	HR	95% CI	*P*
**Age**	1.04	0.98–1.11	0.18	–	–	–
**CAPRA-S**						
Low	Ref.			Ref		
Intermediate	13.8	1.83–104.0	0.01	13.5	1.79–101.4	0.01
High	40.2	5.36–300.9	0.0003	34.8	4.64–261.5	0.0006
**Statin-status**						
Non-user	Ref.			Ref.		
All-users	2.17	1.03–4.58	0.04	1.74	0.74–3.7	0.15
**Plasma-LDL**						
> 3 mmol/L	Ref.					
< 3 mmol/L	0.83	0.41–1.65	0.58	–	–	–
**LDLR expression**						
Low expression	Ref.			Ref.		
High expression	0.46	0.23–0.88	0.019	0.47	0.24–0.94	0.03

BCR: Biochemical recurrence; HR: Hazard Ratio; CI: Confidence Interval; LDLR: Low-density Lipoprotein Receptor; LDL: low-density lipoprotein.

Cox regression analysis. Crude HR and HR adjusted for Capra-S risk group and statin-status. Patients with missing information regarding BCR, time to BCR, total follow-up, or capra_s_risk_group were excluded from the analysis.

Notably, high intra-tumour expression of LDLR was associated with significantly better BCR-free survival compared with low LDLR expression (hazard ratio [HR] = 0.46, 95% confidence interval [CI] = 0.23–0.88, Table 4), and this remained significant also after adjustment for CAPRA-S risk group and statin-user status (HR = 0.47, 95% CI = 0.24–0.94, Table 4).

Moreover, high LDLR expression was significantly associated with longer BCR-free survival in statin users (*p* = 0.02, Figure S6), but not in non-users (*p* = 0.12, Figure S6).

## Discussion and conclusion

In this study, we performed genome-wide expression profiling of tumour samples from a cohort of nearly 200 PC patients (statin-users vs. non-users), to explore possible links between statin use and the prostate tumour transcriptional profile as well as more favourable PC pathobiology. Firstly, we found that gene expression patterns associated with statin use were limited to PC tissue samples, while no significant DEGs were observed in AN tissue in this study. We unveiled a total of 163 DEGs in PC samples from patients who used statin treatment compared with non-users.

Several of the top DEGs have previously been associated with dysregulation in other cancer forms (Table S2), while five downregulated DEGs (*RBPJ, FOXO1, TSC22D3, SNHG6, DEK*) and two upregulated DEGs (*OTUB1, LDLR*) have been explored concerning PC specifically. The specific role of these genes concerning PC is however, not fully understood. Conceivably, based on the existing literature, *RBPJ, SNHG6,* and *DEK* may contribute tumour-suppressive effects following statin use [17–19], while *FOXO1, TSC22D3,* and *OTUB1* may mediate tumour-promoting effects after statin use [20–22]. However it is possible that some of the identified DEGs reflect PC molecular heterogeneity rather than statin treatment itself.

Secondly, we discovered 16 downregulated molecular pathways in PC samples from patients who used statin treatment compared with non-users – most significantly affecting genes in the pathway leading to EMT. Epithelial-mesenchymal transition is a biological process, which is known to initiate tumour invasiveness and metastasis [23]. Therefore, statins could potentially protect against advanced disease by a general decrease in the transcription of genes leading to EMT. Downregulation of EMT has previously been reported in PC cells treated with atorvastatin *in vitro* [24], and our results indicate that this is also the case in human PC tissue.

We explored alterations in cholesterol metabolism and LDLR expression in statin-users vs. non-users in PC tissue. We observed that statin treatment was associated with upregulation of the LDLR, suggesting that statin therapy could affect PC tissue in a fashion similar to the mechanism known from the liver [25]. In support of our findings, a previous study showed decreased intracellular cholesterol levels and increased LDLR expression in PC cell lines treated with simvastatin *in vitro* [26]. Moreover, a randomised-controlled trial from 2019 revealed that atorvastatin accumulates in the prostate as intraprostatic concentrations are elevated compared with plasma concentrations [27]; however, the mechanism by which statins cross cell membranes in the prostate remains unknown.

When exploring statin-mediated effects on other key genes in cholesterol metabolism, we exclusively found downregulation of *CYP27A*1. CYP27A1 converts cholesterol into 27-hydroxycholesterol (27-HC), and is linked to cell proliferation and metastasis in PC [28]. We are not able to clarify the regulatory mechanism leading to LDLR upregulation in statin-users on a transcriptional level, and further mechanistic studies would be highly relevant. We do, however, note that LDLR expression may be linked to AR signalling, as *NCOR1* and *NCOR2* were both negatively correlated with LDLR expression in statin-users.

Low-density Lipoprotein Receptor expression was significantly higher in atorvastatin users compared with simvastatin users, which may suggest that this drug is more efficient when targeting PC.

Interestingly, we showed that LDLR upregulation was associated with early BCR in multivariate analysis; this observation suggests that the protective effect of statins observed in previous PC studies may be mediated by an increase in LDLR transcription in the tumour. The potential protective effect of LDLR upregulation was only present in statin-users, which supports the hypothesis of a statin-mediated effect on PC outcomes.

One potential limitation of this study is the lack of matching baseline characteristics between users and non-users. Table 1 shows that statin-users were older and had a higher CAPRA-S score at the time of RP. Hence, at least some of the observed DEGs between statin-users and non-users might be influenced by potential confounders, warranting further independent validation. The observed age difference between the two groups is likely due to statin treatment being more frequently prescribed to older individuals with age-related diseases [29]. The difference in CAPRA-S score between the two groups may stem from the process of selecting patients suitable for RP, where life expectancy and clinicopathological characteristics influence whether surgery is offered to a patient.

On the other hand, atorvastatin and simvastatin-users, have very similar baseline characteristics. The high expression of the LDLR in atorvastatin vs. simvastatin users would therefore not be influenced by any known confounders. Furthermore, LDLR upregulation between statin-users and non-users was not associated with any clinicopathological characteristics, which supports the hypothesis that this finding is mediated by statin treatment and not confounding variables. In addition, high expression of LDLR was associated with prolonged time to BCR after adjusting for the CAPRA-S risk group.

This study focused on transcriptomics, but future work should assess LDLR protein expression in PC tissue (e.g. via IHC), as done successfully in a prior breast cancer trial [30].

Overall, we revealed 163 genes and 16 pathways in PC tissue that could potentially be associated with the reported protective effect of statins observed in large epidemiological studies [5, 29]. Specifically intra-tumour LDLR upregulation was associated with both statin use and prolonged time to BCR and could play a protective role. Further clinical validation is warranted.

## Supplementary Material



## Data Availability

Available at: https://genome.au.dk/library/GDK000017/
