# Supplementary material for: Up-regulation of intra-tumour LDLR gene expression is associated with statin treatment and better prostate cancer prognosis
Source: Acta Oncol. 2025 Oct 7;64:43788. doi: 10.2340/1651-226X.2025.43788 (PMC12517209; doi:10.2340/1651-226X.2025.43788)
Supplement: Supplementary file 1 [file AO-64-43788-s1.pdf]

Supplementary material has been published as submitted. It has not been copyedited, or typeset by Acta Oncologica.

Table S1: Patient clinical characteristics

	Non-user	Statin-user	Atorvastatin-user	Simvastatin- user
**Patients**				
n	93	93	45	48
**Statin dose**				
Median, mg/day	NA	30.3	32.6	28.5
(IQR)		(20.4-38.0)	(19.4-41)	(20.0-36)
Unknown		0 (0%)	0 (0%)	0 (0%)
**Treatment length**				
Median, years	NA	8.7	9.1	5.8
(IQR)		(4.5- 11.9)	(5.0-11.7)	(3.2-11.5)
Unknown		0 (0%)	0 (0%)	0 (0%)
**P-cholesterol level**				
< 5 mmol/L	33 (35.5%)	70 (75.2%)	31 (68.9%)	39 (81.2 %)
> 5 mmol/L	49 (52.7 %)	19 (20.4%)	10 (22.2%)	9 (18.8%)
Unknown	11 (11.8%)	4 (4.3%)	4 (8.8%)	0 (0%)
**P-LDL level**				
< 3 mmol/L	30 (32.2 %)	68 (73.1%)	31(68.8%)	37 (77.1%)
> 3 mmol/L	49 (52.7 %)	14 (15.1%)	7(15.6%)	7(14.6%)
Unknown	14 (18.3 %)	11 (11.8%)	7(15.6%)	4(8.3%)

Data is n (%) or median (IQR).
Table text: IQR: interquartile range; NA: not applicable

Figure S1: Sample and data filtration

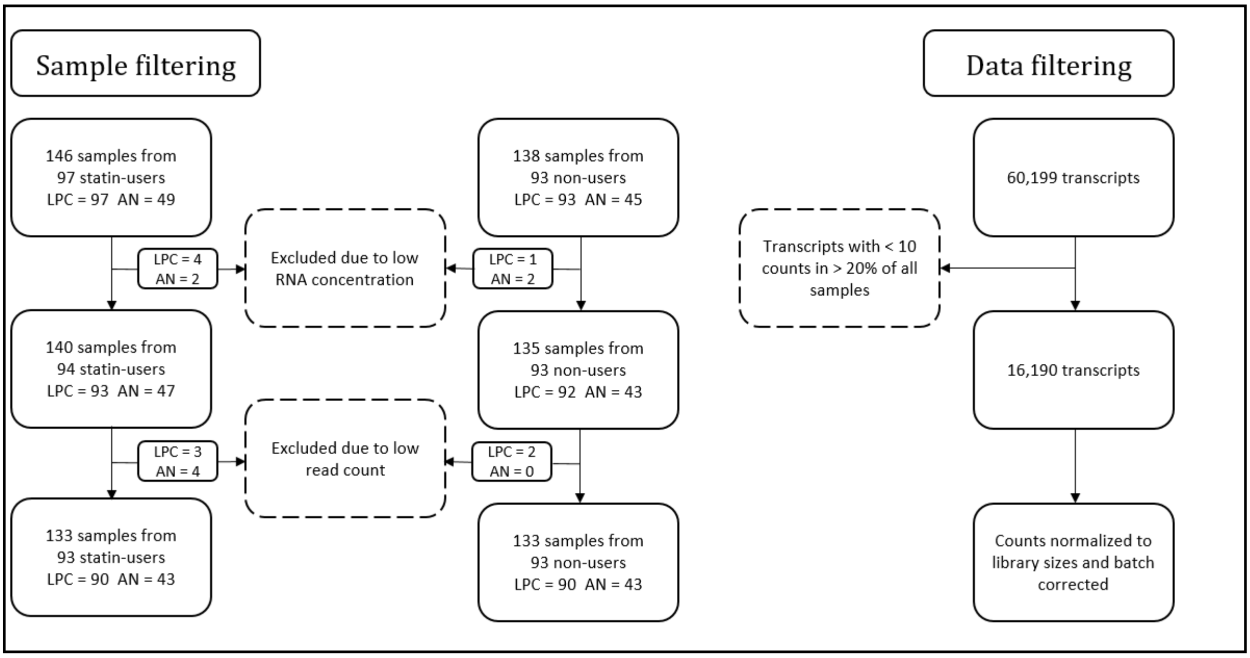

Figure text: LPC: Localized prostate cancer tissue; AN: Adjacent nonmalignant prostate tissue

Table S2: Differential expression analysis

Gene	P.val	P.adj	LogFC	Nomenclature	Role
**Top 30 differentially expressed genes**					
**Downregulated in statin-users vs. non-users**					
**Gene**	**P.val**	**P.adj**	**LogFC**	**Nomenclature**	**Role**
**ZNF853**	5.93E-08	0.001	-0.870	Zinc Finger Protein 853	Transcription factor (RNA binding), not previously associated with cancer (1).
**DCAF12L2**	1.43E-06	0.006	-1.123	DDB1 And CUL4 Associated Factor 12 Like 2	Involved in a variety of cellular processes. High expression prolongs overall survival in breast cancer (2).
**ZNF800**	1.48E-06	0.006	-0.291	Zinc Finger Protein 800	DNA binding. A high expression is associated with lung cancer(3) and colorectal cancer (4).
**NEURL1B**	2.72E-06	0.009	-0.545	Neuralized E3 Ubiquitin Protein Ligase 1B	Protein degradation and endocytosis. Downregulated in colorectal cancer (5).
**RBPJ**	3.33E-06	0.009	-0.191	Recombination Signal Binding Protein For IG Kappa J Region	Transcriptional regulator in the Notch signaling pathway. Has tumor-promoting capacities (6).
**FOXO1**	4.72E-06	0.011	-0.451	Forkhead Box O1	PI3K/AKT signaling. Described as a tumor suppressor in PC cancer (7, 8).
**TSC22D3**	1.42E-05	0.023	-0.493	TSC22 Domain Family Member 3	Anti-inflammatory (9). Expression change with tumor grade/stage in PC (10).
**SNHG6**	1.54E-05	0.023	-0.336	Small Nucleolar RNA Host Gene 6	Long non-coding RNA. Overexpressed in PC and associated with shorter disease-free survival.(11)
**DOCK11**	1.59E-05	0.023	-0.740	Dedicator Of Cytokinesis 11	B cell differentiation. Overexpression linked to testicular carcinoma. (12)
**PPP4R2**	2.86E-05	0.027	-0.223	Protein Phosphatase 4 Regulatory Subunit 2	DNA double strand break repair. Low expression associated with leukemia. (13)
**CTDSPL**	3.16E-05	0.027	-0.251	CTD Small Phosphatase Like	Regulator of cell cycle. Described as tumor suppressor in lung cancer. (14)
**AL445426.1**	4.18E-05	0.029	-0.962	-	-
**DEK**	5.21E-05	0.030	-0.199	DEK Proto-Oncogene	DNA modulation. Overexpression is associated with PC progression in neuroendocrine PC (15).
**COL17A1**	5.98E-05	0.030	-1.282	Collagen Type XVII Alpha 1 Chain	Transmembrane alpha chain collagen. Overexpression is seen in pancreas cancer(16), but in the contrary related to prolonged overall survival in breast cancer(17).

**Upregulated in statin-users vs. non-users**					
**AC073225.1**	1.07E-06	0.006	0.787	-	-
**CCDC124**	6.56E-06	0.013	0.401	Coiled-Coil Domain Containing 124	RNA binding, is overexpressed in several cancers (18).
**PGBD4**	1.9E-05	0.026	0.510	PiggyBac Transposable Element Derived 4	Transposase(1). Not previously associated with cancer
**GOLGA6L2**	2.29E-05	0.027	1.343	Golgin A6 Family Like 2	A part of the Protein receiving Cis-Golgi (1). Not previously associated with cancer.
**COA7**	2.75E-05	0.027	0.467	Cytochrome C Oxidase Assembly Factor 7	Mitochondrial respiration (1). Not previously associated with cancer.
**RNF223**	2.85E-05	0.027	0.965	Ring Finger Protein 223	Protein posttranslational modification. Downregulated is a unfavorable prognostic marker in Pancreas cancer(19)
**AL354919.1**	2.9E-05	0.027	0.590	-	-
**AP002748.6**	3.03E-05	0.027	0.973	-	-
**MIR3936HG**	3.34E-05	0.027	0.594	MIR3936 Host Gene	lncRNA(1). Not previously associated with cancer.
**BCL2L14**	3.69E-05	0.027	0.778	BCL2 Like 14	Apoptotic regulator. Overexpressed in breast cancer(20).
**MIEF2**	3.7E-05	0.027	0.412	Mitochondrial Elongation Factor 2	Regulates mitochondrial morphology. Overexpressied in ovarian cancer(21). Low expression observed in colon cancer and related to oxaliplatin resistance(22).
**OR7E122P**	4.47E-05	0.030	1.083	Olfactory Receptor Family 7 Subfamily E Member 122	G protein-mediated transduction (1). Not previously associated with cancer.
**OTUB1**	5.09E-05	0.030	0.242	OTU Deubiquitinase, Ubiquitin Aldehyde Binding 1	Protein turnover. Overexpressed in PC(23, 24).
**RN7SL440P**	5.21E-05	0.030	0.886	-	-
**LDLR**	5.37E-05	0.030	0.416	Low Density Lipoprotein Receptor	LDL/cholesterol uptake. LDLR knock-down inhibits growth in PC cell lines (25). PC cells shown to lack normal feedback regulation of LDLR expression (26).
**GRHPR**	6.02E-05	0.030	0.239	Glyoxylate And Hydroxypyruvate Reductase	Metabolism. High expression prolongs overall survival in breast cancer(27).

Table text: P.val: p-value; P.adj: BH adjusted p-value; LogFC: log2 fold change
Differential expression analysis with top DEGs between statin-users and non-users in PC samples based on P-value.

Table S3: Androgen Receptor signaling and LDLR expression

Gene	Median expression level (LogCPM) All patients (n =180)			Correlation with LDLR expression Non-users (n = 90)		Correlation with LDLR expression Statin-users (n = 90)		Nomenclature
	Statin-users	Non-users	P. val.	R	P. val.	R	P. val.	
**AR**	7.96	8.11	0.1	-0.2	0.06	-0.19	0.07	Androgen receptor
**KLK3**	12.26	12.16	1	-0.19	0.08	-0.042	0.69	Prostate Specific Antigen
**NCOA1**	5.97	5.96	1	-0.074	0.49	-0.076	0.47	Nuclear receptor coactivator 1
**NCOA2**	4.86	4.94	0.18	-0.14	0.2	-0.062	0.56	Nuclear receptor coactivator 2
**NCOA3**	5.59	5.6	0.88	-0.21	0.05	-0.08	0.46	Nuclear receptor coactivator 3
**NCOR1**	8.1	8.1	0.45	-0.16	0.13	-0.3	0.004	Nuclear receptor corepressor 1
**NCOR2**	7.04	7.13	0.026	-0.3	0.005	-0.4	<0.0001	Nuclear receptor corepressor 2

Table text: LogCPM: Log counts pr. Million; P.val: p value; R: Spearman correlation coefficient Expression level of genes described in androgen receptor signaling statin-users vs. non-users compared using Wilcoxon signed-rank. The correlation between gene expression and LDLR expression was assessed using Spearman correlation.

Table S4: Genes contributing with the highest rank in EMT downregulation

Gene	RankMetric	Median expression level (LogCPM)			Role
		Statin-users	Non-users	P. val.	
**GAS1**	-0.66	2.4	2.98	0.008	Acts as a tumor suppressor in colorectal cancer. Is expression increases E-cadherin and decreases Snail and N-cadherin in cancer cells.
**ACTA2**	-0.63	9.17	9.68	0.005	Encodes alpha-smooth muscle actin, a hallmark of the mesenchymal/myofibroblast phenotype; upregulated during EMT and involved in prostate cancer stroma remodeling.
**FLNA**	-0.52	9.58	10.03	0.001	Actin-binding cytoskeletal protein that supports cell motility and structural reorganization; plays a role in EMT and metastasis.
**MYLK**	-0.48	8.52	8.9	0.004	Regulates actomyosin contraction and cell migration; contributes to cytoskeletal changes required for EMT.

Table text: LogCPM: Log counts pr. Million; P.val: p value
Expression level of Leading-Edge genes (RankMetric) for the Hallmark EMT pathway analysis. Statin-users vs. non-users are compared using the Wilcoxon signed-rank.

Figure S2: Principal Component Analysis

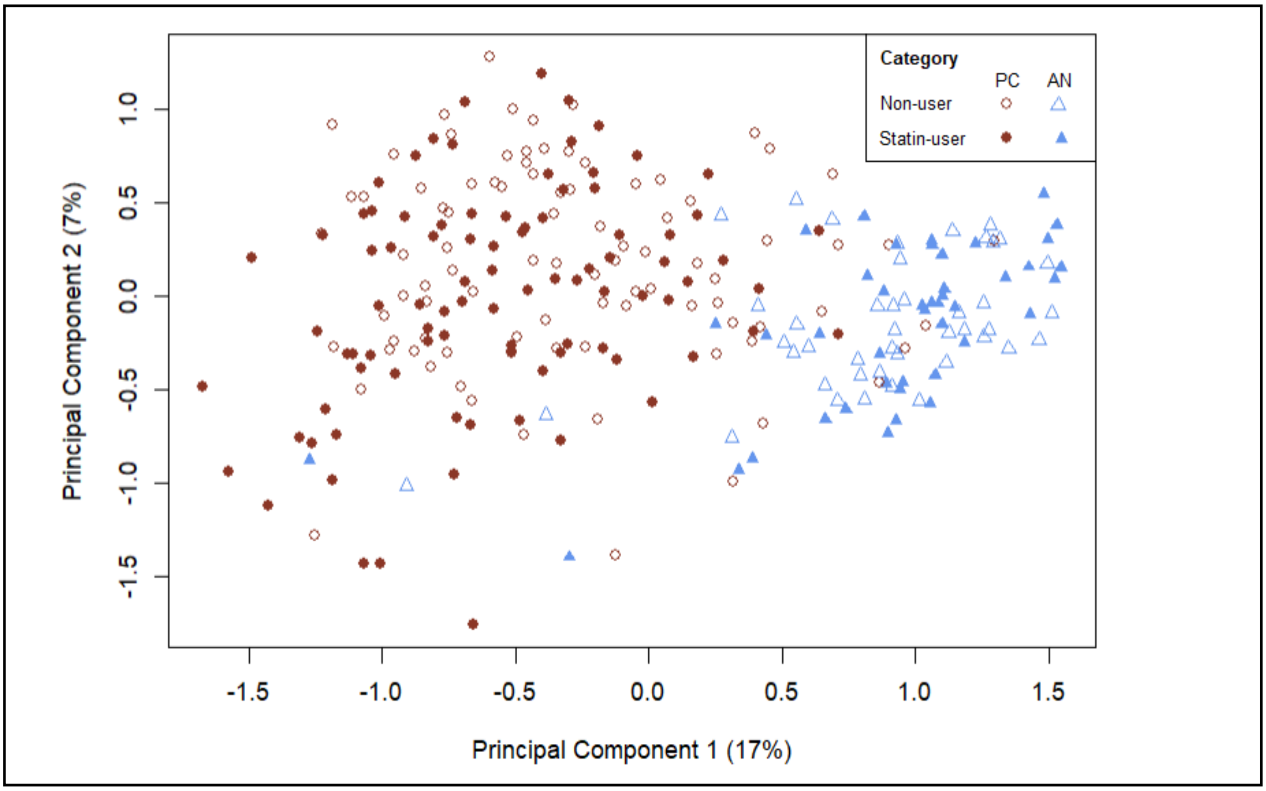

Figure text: PC: Prostate Cancer tissue; AN: Adjacent nonmalignant tissue; LogFC: log2 fold-change.

Unsupervised analysis illustrating the greatest variance among all 16,190 genes.

Figure S3: Volcano plot illustrating all DEGs between PC and AN tissue

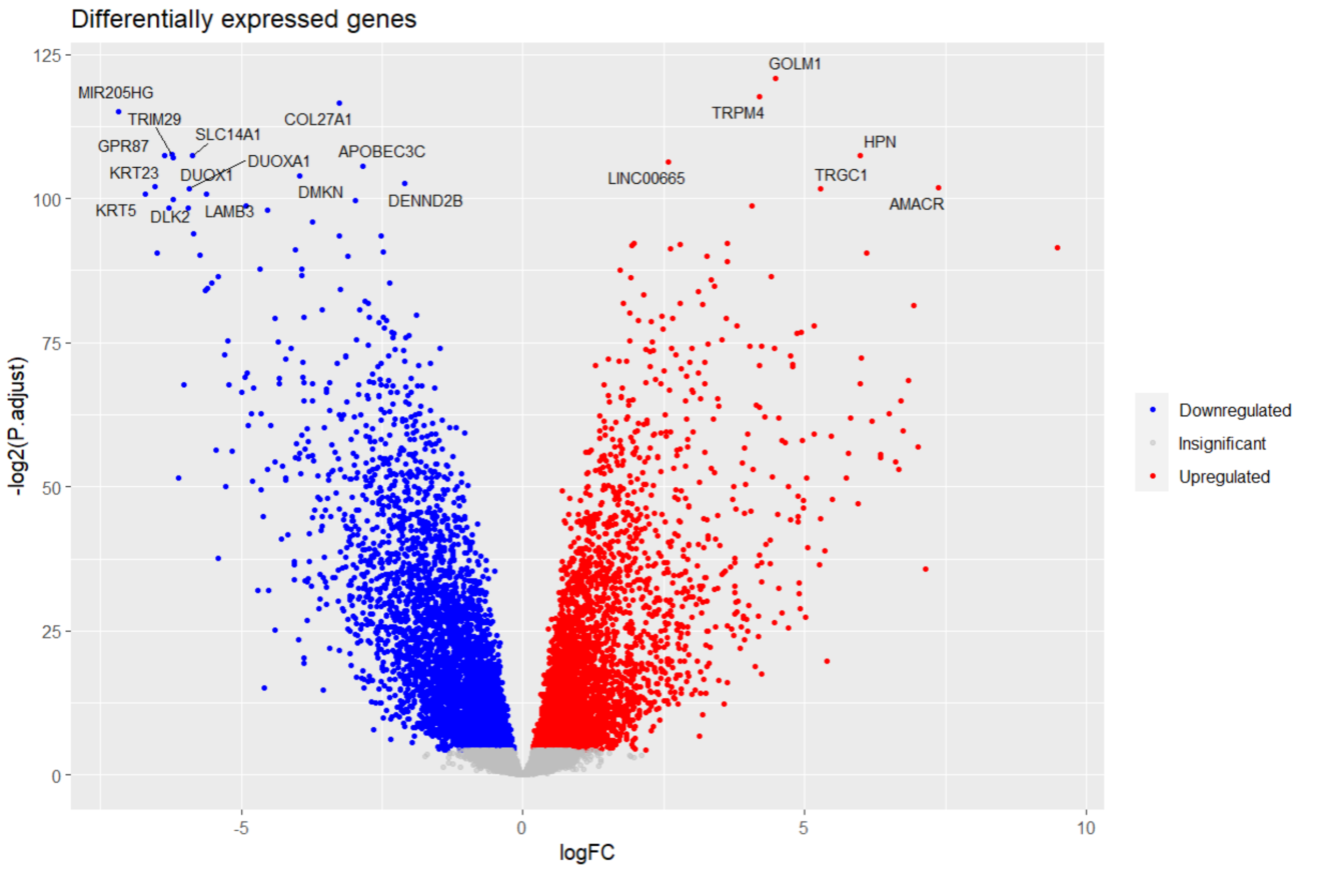

Figure text: DEGS: Differentially expressed genes; PC: Prostate Cancer tissue; AN: Adjacent nonmalignant tissue; LogFC: log2 foldchange p.adjust: Adjusted p-values
Label on top 20 DEGs based on p-value.

Figure S4: Gene set enrichment analysis (GSEA) between statin-users and non-users

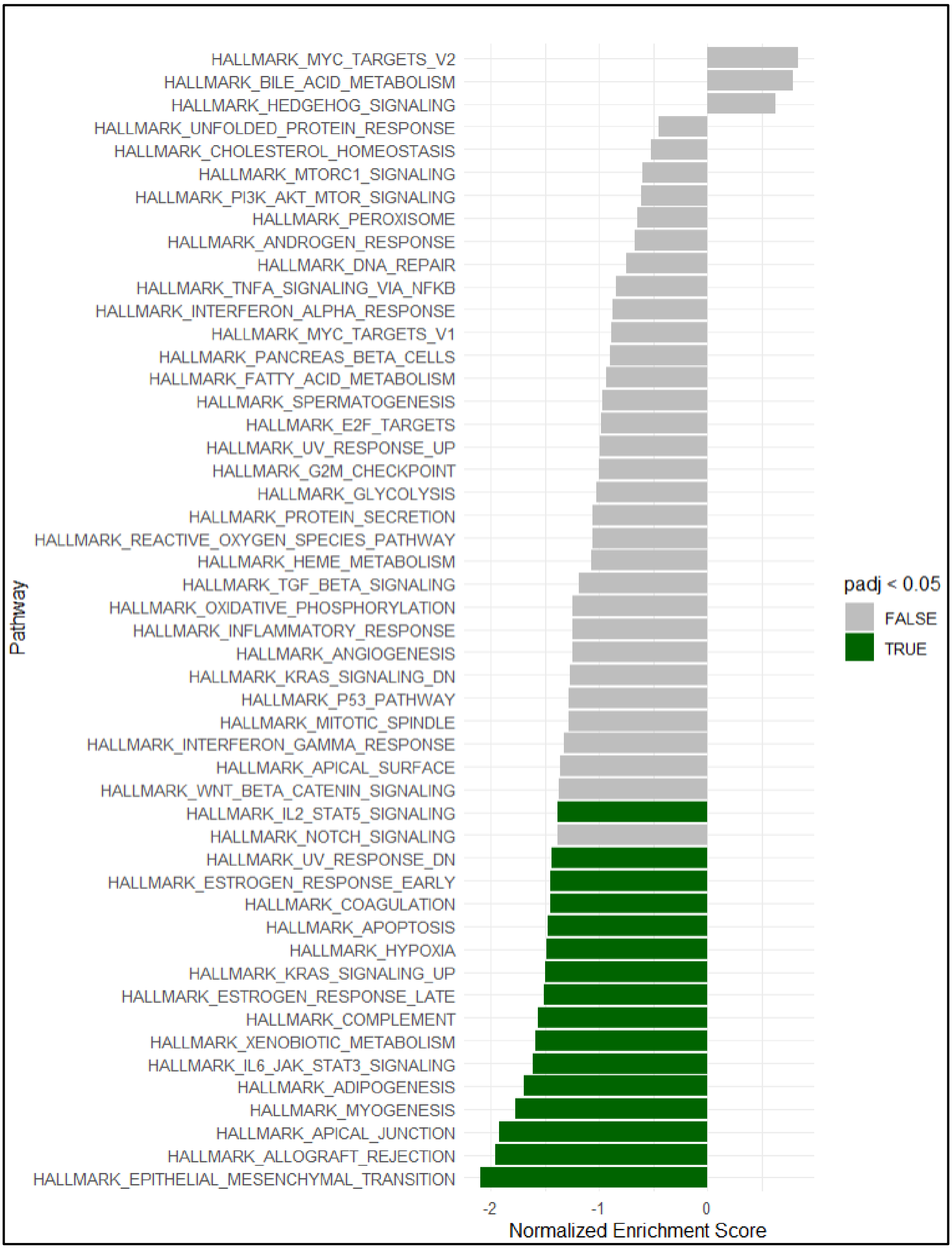

Figure text: P.adj: adjusted p-value

Hallmark gene sets enrichment analysis based on all 16,190 genes in statin-users versus non-users in PC samples. Green bars are significantly downregulated pathways in statin-users versus non-users. No pathways were significantly upregulated. Significance threshold = p-value < 0.05, adjusted by the Benjamin-Hochberg Procedure.

Figure S5: LDLR expression association with patient clinical characteristics

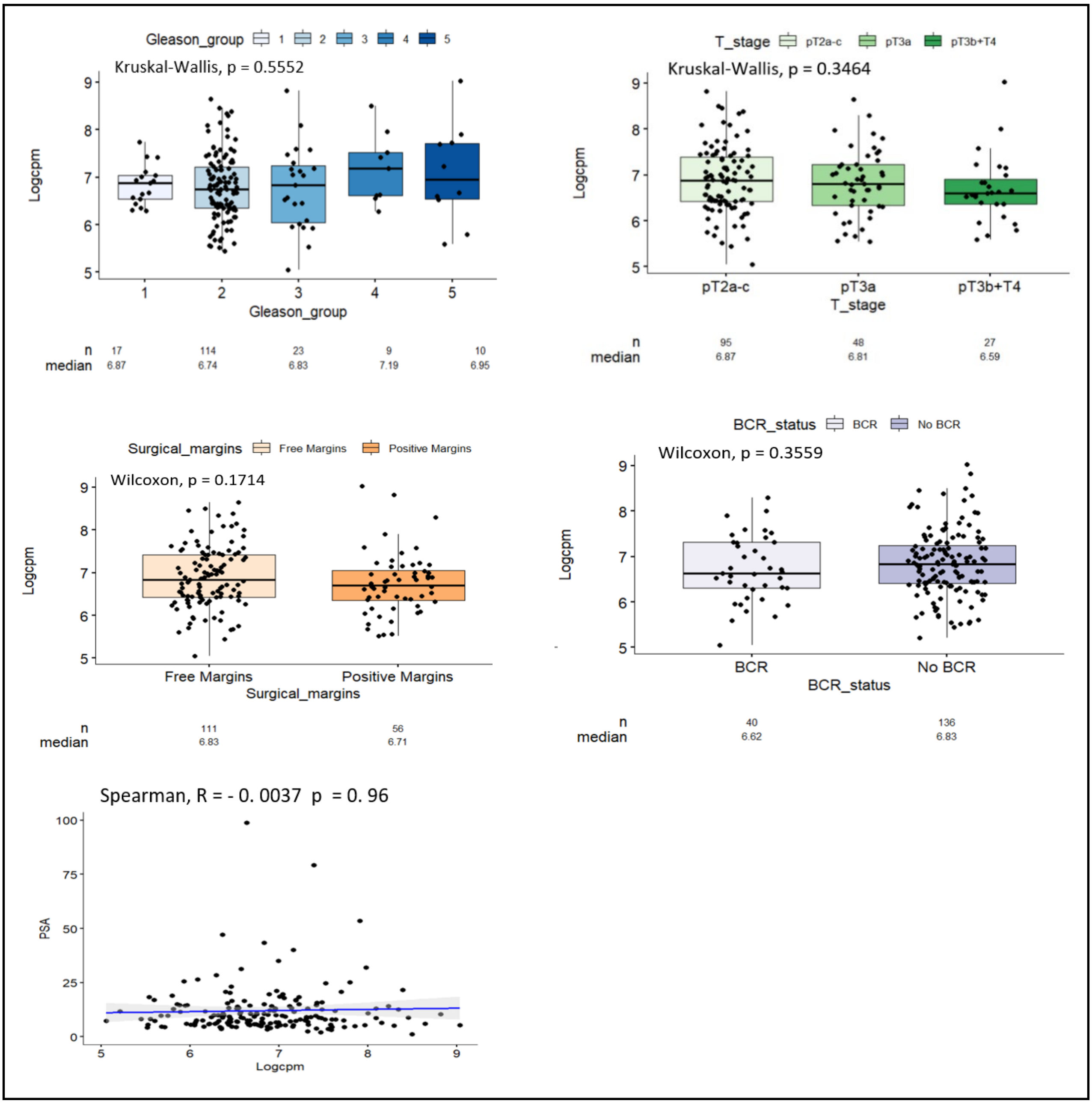

Figure text: P: p-value; BCR: Biochemical recurrence; R: Correlation coefficient; IQR: Interquartile range.

Association of LDLR expression to GG, pT-stage, surgical margin status, BCR status and PSA level before RP.

Figure S6: Kaplan-Meier analysis of BCR time in statin-users and non-users

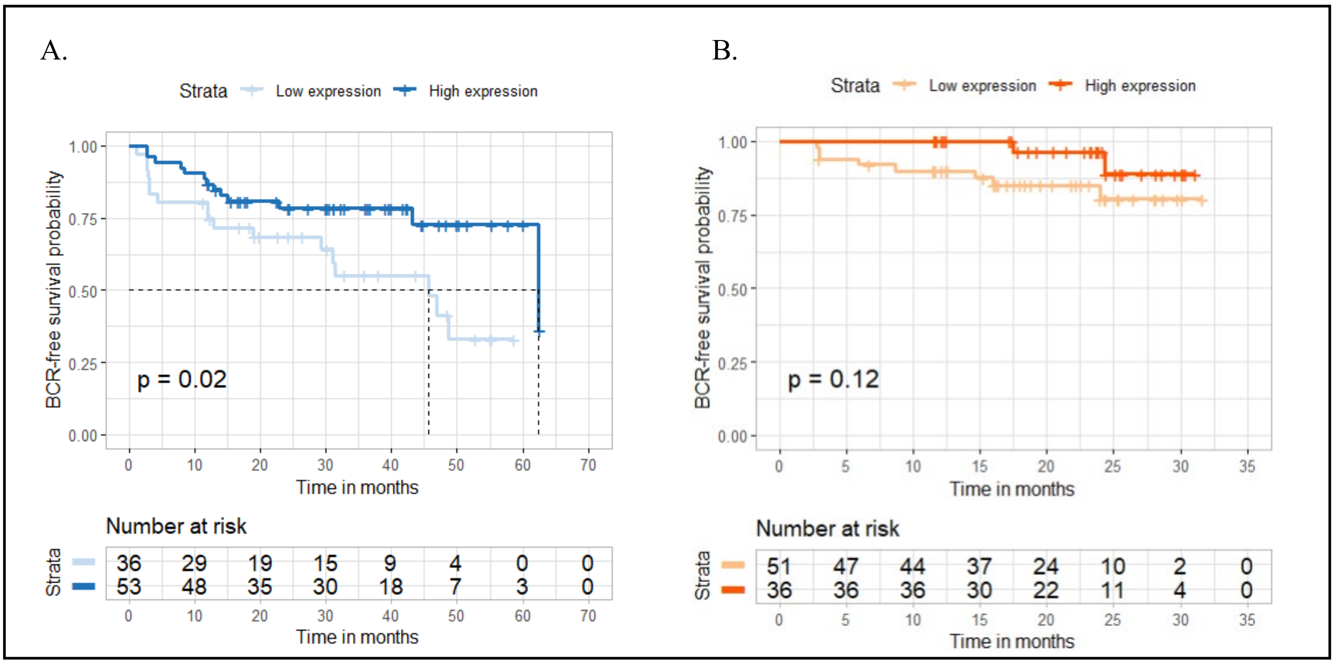

Figure text: P: p-value; BCR: Biochemical recurrence.
Kaplan-Meier analysis of BCR-free survival with low and high LDLR expression in statin-users (A) and non-users (B). Patients with missing information regarding BCR, time to BCR, or total follow-up time were excluded from this analysis.

Supplementary References

1. Stelzer G, Rosen N, Plaschkes I, Zimmerman S, Twik M, Fishilevich S, et al. The GeneCards Suite: From Gene Data Mining to Disease Genome Sequence Analyses. Curr Protoc Bioinformatics. 2016;54:1.30.1-1..3.
2. Zhang XX, Yu X, Zhu L, Luo JH. Establishment of a 6-signature risk model associated with cellular senescence for predicting the prognosis of breast cancer. Medicine (Baltimore). 2023;102(46):e35923.
3. Zhuo E, Cai C, Liu W, Li K, Zhao W. Downregulated microRNA-140-5p expression regulates apoptosis, migration and invasion of lung cancer cells by targeting zinc finger protein 800. Oncol Lett. 2020;20(6):390.
4. Rengganaten V, Huang CJ, Wang ML, Chien Y, Tsai PH, Lan YT, et al. Circular RNA ZNF800 (hsa_circ_0082096) regulates cancer stem cell properties and tumor growth in colorectal cancer. BMC Cancer. 2023;23(1):1088.
5. Liu J, Liu Z, Zhang X, Yan Y, Shao S, Yao D, et al. Aberrant methylation and microRNA-target regulation are associated with downregulated NEURL1B: a diagnostic and prognostic target in colon cancer. Cancer cell international. 2020;20:342.
6. Xue L, Li H, Chen Q, Wang Z, Zhang P, Chen H, et al. Inhibition of Recombining Binding Protein Suppressor of Hairless (RBPJ) Impairs the Growth of Prostate Cancer. Cell Physiol Biochem. 2015;36(5):1982-90.
7. Zhao Y, Tindall DJ, Huang H. Modulation of androgen receptor by FOXA1 and FOXO1 factors in prostate cancer. Int J Biol Sci. 2014;10(6):614-9.
8. Yan Y, Huang H. Interplay Among PI3K/AKT, PTEN/FOXO and AR Signaling in Prostate Cancer. Adv Exp Med Biol. 2019;1210:319-31.
9. Ding S, Pang X, Luo S, Gao H, Li B, Yue J, et al. Dynamic RBM47 ISGylation confers broad immunoprotection against lung injury and tumorigenesis via TSC22D3 downregulation. Cell Death Discov. 2023;9(1):430.
10. Munkley J, Maia TM, Ibarluzea N, Livermore KE, Vodak D, Ehrmann I, et al. Androgen-dependent alternative mRNA isoform expression in prostate cancer cells. F1000Res. 2018;7:1189.
11. Yan Y, Chen Z, Xiao Y, Wang X, Qian K. Long non-coding RNA SNHG6 is upregulated in prostate cancer and predicts poor prognosis. Mol Biol Rep. 2019;46(3):2771-8.
12. Almstrup K, Leffers H, Lothe RA, Skakkebaek NE, Sonne SB, Nielsen JE, et al. Improved gene expression signature of testicular carcinoma in situ. Int J Androl. 2007;30(4):292-302; discussion 3.
13. Herzig JK, Bullinger L, Tasdogan A, Zimmermann P, Schlegel M, Teleanu V, et al. Protein phosphatase 4 regulatory subunit 2 (PPP4R2) is recurrently deleted in acute myeloid leukemia and required for efficient DNA double strand break repair. Oncotarget. 2017;8(56):95038-53.
14. Krasnov GS, Puzanov GA, Afanasyeva MA, Dashinimaev EB, Vishnyakova KS, Beniaminov AD, et al. Tumor suppressor properties of the small C-terminal domain phosphatases in non-small cell lung cancer. Biosci Rep. 2019;39(12).
15. Lin D, Dong X, Wang K, Wyatt AW, Crea F, Xue H, et al. Identification of DEK as a potential therapeutic target for neuroendocrine prostate cancer. Oncotarget. 2015;6(3):1806-20.

16. Yang J, Li Y, Sun Z, Fan Z, Shi M, Liu S, et al. *COL17A1 facilitates tumor growth and predicts poor prognosis in pancreatic cancer. Biochem Biophys Res Commun. 2022;632:1-9.*

17. Du F, Zheng F, Han Y, Zhao J, Yuan P. *Novel Immune-Related Gene Signature for Risk Stratification and Prognosis of Survival in ER (+) and/or PR (+) and HER2 (-) Breast Cancer. Front Pharmacol. 2022;13:820437.*

18. Arslan Ö, Soylu NK, Akillilar PT, Tazebay UH. *Coiled-coil domain-containing protein-124 (Ccdc124) is a novel RNA binding factor up-regulated in endometrial, ovarian, and urinary bladder cancers. Cancer Biomark. 2021;31(2):149-64.*

19. Feng L, Wang J, Zhang J, Diao J, He L, Fu C, et al. *Comprehensive Analysis of E3 Ubiquitin Ligases Reveals Ring Finger Protein 223 as a Novel Oncogene Activated by KLF4 in Pancreatic Cancer. Front Cell Dev Biol. 2021;9:738709.*

20. Romero P, Benhamo V, Deniziaut G, Fuhrmann L, Berger F, Manié E, et al. *Medullary Breast Carcinoma, a Triple-Negative Breast Cancer Associated with BCLG Overexpression. Am J Pathol. 2018;188(10):2378-91.*

21. Zhao S, Zhang X, Shi Y, Cheng L, Song T, Wu B, et al. *MIEF2 over-expression promotes tumor growth and metastasis through reprogramming of glucose metabolism in ovarian cancer. J Exp Clin Cancer Res. 2020;39(1):286.*

22. Xie C, Li K, Li Y, Peng X, Teng B, He K, et al. *CRISPR-based knockout screening identifies the loss of MIEF2 to enhance oxaliplatin resistance in colorectal cancer through inhibiting the mitochondrial apoptosis pathway. Front Oncol. 2022;12:881487.*

23. Iglesias-Gato D, Chuan YC, Jiang N, Svensson C, Bao J, Paul I, et al. *OTUB1 de-ubiquitinating enzyme promotes prostate cancer cell invasion in vitro and tumorigenesis in vivo. Mol Cancer. 2015;14(1):8.*

24. Liao Y, Wu N, Wang K, Wang M, Wang Y, Gao J, et al. *OTUB1 Promotes Progression and Proliferation of Prostate Cancer via Deubiquitinating and Stabling Cyclin E1. Front Cell Dev Biol. 2020;8:617758.*

25. Furuya Y, Sekine Y, Kato H, Miyazawa Y, Koike H, Suzuki K. *Low-density lipoprotein receptors play an important role in the inhibition of prostate cancer cell proliferation by statins. Prostate Int. 2016;4(2):56-60.*

26. Hughes-Fulford M, Chen Y, Tjandrawinata RR. *Fatty acid regulates gene expression and growth of human prostate cancer PC-3 cells. Carcinogenesis. 2001;22(5):701-7.*

27. Wang J, Wang Y, Xing P, Liu Q, Zhang C, Sui Y, et al. *Development and validation of a hypoxia-related prognostic signature for breast cancer. Oncol Lett. 2020;20(2):1906-14.*
